# Epidemiology of hepatitis A in Greece in the last decade: management of reported cases and outbreaks and lessons learned

**DOI:** 10.1017/S0950268820000382

**Published:** 2020-02-13

**Authors:** K. Mellou, A. Chrysostomou, T. Sideroglou, M. Kyritsi, T. Georgakopoulou, S. Tsiodras, C. Hadjichristodoulou

**Affiliations:** 1 Department of Epidemiological Surveillance and Intervention, Hellenic Public Health Organization, Athens, Greece; 2 Regional Public Health Laboratory (PEDY) of Thessaly, Thessaly, Greece; 3 Department of Hygiene and Epidemiology, Medical School, University of Thessaly, Larissa, Greece; 4 4th Academic Department of Medicine, National and Kapodistrian University of Athens, Medical School, Athens, Greece

**Keywords:** Epidemiology, hepatitis A, outbreaks, public health, vaccination (immunisation)

## Abstract

Hepatitis A is a mandatory notifiable disease in Greece. Here, we present the epidemiological data for 2009–2018 and the results of outbreak investigations performed, and discuss future public health priorities.

Overall, 1193 cases were reported; 320 migrants/refugees, 240 Roma, 112 travellers and 521 from the general population. The median age of the affected general population (37 years) had an increasing trend (from 30.8 years in 2009 to 40.5 in 2018, *P* < 0.001) and was significantly higher than that among Roma and migrants (7 and 8 years, respectively, *P* < 0.001). Twenty-two cases (2.2%) were unvaccinated patients with a chronic liver disease. Fifty clusters with 2–12 cases each were recorded; 44 were attributed to person-to-person transmission and six to food consumption. Three outbreaks accounting for 32.3% of the total number of recorded cases were identified; in 2013 among Roma (112 cases), in 2016 among refugees (188 cases) and in 2017 among men having sex with men (96 cases; 33 of them (34.4%) HIV-positive). The epidemiological data depict that improving living conditions and vaccination coverage of deprived populations, and informing adults on the disease focusing at faecal–oral transmission during sexual intercourse and travel should be the future public health priorities.

## Introduction

Hepatitis A is an acute liver disease caused by the hepatitis A virus (HAV). HAV infection typically causes a self-limited illness with jaundice that is usually mild or can even be asymptomatic. Although uncommon, severe hepatic and extrahepatic complications, including liver failure, may also occur. Improvement of hygienic standards and sanitation in a country is followed by a decreased incidence of the disease, and a safe, cheap and effective vaccine against HAV is available [1].

According to the latest reported data of the European Centre for Disease Prevention and Control, the mean notification rate reported by the European Union and European Economic Area (EU/EEA) countries was 3.03 cases per 100 000 population for the year 2018 [2]. Overall, data indicate that even though the notification rate in Europe is low, the disease occurs at higher ages compared to the past, increasing the chance for potentially large outbreaks in older non-immunised individuals [3]. In 2017, notification rate was doubled compared to 2016 mainly due to the occurrence of a large European HAV outbreak disproportionately affecting men who have sex with men (MSM) [4].

In Greece, the notification rate of HAV disease substantially declined in the 80s due to the improvement of the socio-economic conditions [5, 6]. The vaccine against HAV was first licensed in 1999, and in 2008, it was introduced to the routine National Immunization Program for all children older than 12 months (NIPC; two doses with a time-lag of 6 months) and is fully reimbursed. At the time the decision for the inclusion of the vaccine at the NIPC was made, the annual notification rate was quite stable; however, cases in the general population and outbreaks, especially among the Roma population, continued to occur. Moreover, paediatricians had already started vaccinating children on parents' expense [6]. Apart for the childhood population, HAV vaccination is also recommended for high-risk population groups, such as travellers to endemic countries, MSM, persons with a chronic liver disease and persons who anticipate close contact with an international adoptee from a country of high or intermediate endemicity.

Despite these recommendations and the implementation of universal childhood vaccination in the country, contrary to the vaccination policy of the other European countries that do not systematically vaccinate children against HAV, outbreaks continue to occur in Greece.

The aim of this article is to summarise the available epidemiological data of HAV disease in the country over the period 2009–2018, discuss the factors that led to the occurrence of outbreaks at specific groups of the population, and define priorities for future public health action.

## Methods

HAV is included in the Mandatory Notification System in Greece. New cases are notified by clinical doctors and microbiologists working at the public or private sector to the local public health authorities and the Hellenic Public Health Organization simultaneously. Duplications are handled at the central level. The notification is case-based and the reporting form includes information on demographic, clinical and laboratory data, vaccination status, travel and ethnicity. Specific populations at risk for HAV acquisition based on previously available epidemiological data are recorded; (a) the Roma population, (b) migrants (including economic immigrants, asylum seekers and refugees) and (c) travellers to endemic countries and migrants visiting friends and relatives in their country of origin (VFRs). All non-travel-related cases are interviewed and information on food and water consumption history, as well as other possible risk factors, is recorded.

Cases are classified in accordance with the European case definition [7]. A confirmed case is defined as any symptomatic case of acute illness with a discrete onset of any sign or symptom consistent with acute viral hepatitis (e.g. fever, headache, malaise, anorexia, nausea, vomiting, diarrhoea, abdominal pain), and either (i) jaundice, (ii) fever or (iii) elevated serum alanine aminotransferase (ALT or SGPT) or aspartate aminotransferase (AST or SGOT) levels, and tested positive for anti-IgM HAV. Cases that meet the clinical criteria and have an epidemiological link with a laboratory-confirmed case are classified as probable.

In the case of outbreaks, the results of epidemiological, laboratory and environmental investigation are recorded in a specially designed database, including information on the size of the outbreak, the characteristics of the cases (age, gender, population group), geographical distribution, laboratory testing, living conditions and other factors contributing to the occurrence of the outbreak. Management of cases and public measures taken is also recorded in a coded way.

For 2009–2018, data were analysed for the whole population, by population group and by age group (0–4, 5–14, 15–24, 25–44, 45–64, 65+ years). Analysis of data was carried out with STATA version 12 software (Stata Corporation LP, Texas, USA). All statistical tests were two-tailed.

## Laboratory investigation

During large outbreaks, stool samples from confirmed by serology cases were PCR tested for the detection and typing of the VP1-2a region according to the HAV-Net protocol [8].

For the nucleic acid extraction, the iPrep™ Invitrogen Purification Instrument (Thermo Fisher Scientific, USA) with the iPrep PureLink Invitrogen Virus Kit (Thermo Fisher Scientific) was used. A Reverse Transcriptase PCR (RT-PCR) was performed using the Super Script III One-Step qRT-PCR System with the Platinum Taq Polymerase kit (Invitrogen), in the validated Eppendorf Mastercycler Gradient System. Sequencing was performed using an ABI 3730 DNA Analyser and the sequences were identified using the Hepatitis A Virus Genotyping Tool Version 1.0 (https://www.rivm.nl/mpf/typingtool/hav/introduction).

Where inquiries were launched in EPIS-FWD, sequences of the viral strains were aligned using the Clustal Omega software (https://www.ebi.ac.uk/Tools/msa/clustalo/). According to the ECDC recommendations, a sequence of a minimum of 300 nucleotides with 99.3% homology to one of the outbreak strains, based on the overlapping fragment at the VP1-2a region, was considered as a confirmed case. Cases with <99.3% homology were included as probable or possible if fitting the related case definition.

## Results

During the period 2009–2018, 1193 HAV cases were reported; 1159 confirmed and 34 probable. The mean annual notification rate was 1.08 cases per 100 000 population. The lowest and the highest number of cases were recorded in 2011 (41 cases) and 2017 (294 cases), respectively. The median age of cases was 24 years (range 0–85.6) and 780 (65.4%) cases were males.

Vaccination history was available for 971 (81.4%) of the cases. Of them, 25 (2.6%) reported being vaccinated against HAV; nine (36%) migrants, four (16%) Roma cases, three (12%) travel-related cases and nine (36%) persons that did not belong to any of the recorded risk groups. Fourteen of the 25 vaccinated cases reported that they had received a single dose of the vaccine, while information for 11 cases was not available.

Regarding the disease severity, 1020 (85.5%) cases were hospitalised. The median SGOT/AST of the reported cases was 958 U/L (12–11 784 U/L) and the median SGPT/ALT was 1563 U/L (6–10 255 U/L). Out of the 1127 cases that the respective information was available, 961 (85.3%) had jaundice and 73 of the hospitalised cases (6.5%) developed fulminant hepatitis. Data on the outcome of the disease are available for 53 of these cases. No death was recorded during the study period.

## Risk groups for the disease, 2009–2018

**Fig. 1.**
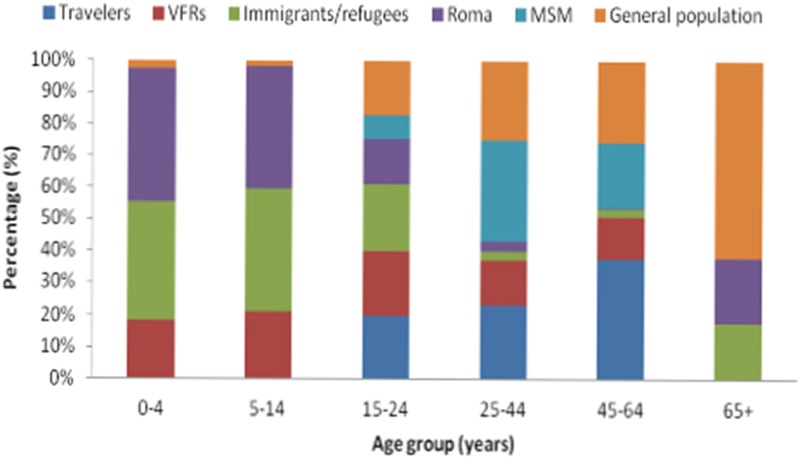
Age distribution of hepatitis A notified cases by population group in travellers, people who visited friends and relatives in their country of origin (VFRs), migrants, Roma, men having sex with men (MSM) and the general population (population after subtracting travellers, migrants and Roma), Mandatory Notification System, Greece, 2009–2018*.

The annual number of reported cases by population group including outbreak cases for the period 2009–2018 is presented in Table 1. As shown in Table 2, the median age of reported cases among Roma and migrants was significantly lower than the median age of cases in the general population (7, 8 and 37 years, respectively, *P* < 0.001). The median age of affected general population ranged from 30.8 years in 2009 to 40.5 years in 2018, showing an increasing trend (*P* < 0.001). The age distribution of HAV cases by population group is depicted in Figure 1. One hundred and ninety-three and two hundred and forty-six (80.7% and 77.6%, respectively) cases among Roma and migrants were children <15 years old, while in the general population, only 21 (5.3%) cases belonged to this age group (*P* < 0.001).

**Table 1.** Annual number of notified cases of hepatitis A by population group^a^, including outbreak cases, Greece, Mandatory Notification System, 2009–2018

Year	Travel-related cases^b^	Migrants^c^	Roma	General population^d^	Total number of reported cases
2009	8	12	9	60	89
2010	2	3	27	26	58
2011	7	3	12	19	41
2012	8	3	25	38	74
2013	8	2	112	43	165
2014	5	12	48	21	86
2015	11	15	6	30	62
2016	7	187	0	20	214
2017	26	30	1	237^e^	294
2018	15	53	0	42	110
Total	97	320	240	536	1193

a The Greek population is estimated at 10 816 286, migrants at 912 000 (census data), refugees hosted in the country since 2016 at around 50 000 [6, 7] and Roma people at 120 000–300 000 people [8].

b People visiting friends and relatives in their country of origin (VFRs) are included.

c Economic immigrants, asylum seekers and refugees are included.

d General population: population after subtraction of travellers, migrants and Roma people.

e Ninety-six of the 237 recorded cases regarded men having sex with men (MSM) (information not recorded systematically in the previous years). Fifteen out of the 96 MSM cases were travel-related.

**Table 2.** Median age and gender of hepatitis A notified cases by population group in Greece, Mandatory Notification System, 2009–2018

	Travel-related cases (*n* = 84)	VFRs^a^ (*n* = 28)	Migrants/refugees (*n* = 320)	Roma (*n* = 240)	MSM^b^ (*n* = 113)	General population (*n* = 408)
Median age (range) in years	39.3 (7–58.9)	21.3 (1.3–54.1)	8 (1–66)	7 (0.3–70)	38.5 (21–55)	37 (0.3–85.6)
Gender						
Male	73.4%	58.1%	60.5%	51.2%		73.9%
Female	26.6%	41.9%	39.5%	48.7%		26.1%

a People visiting friends and relatives in their country of origin.

b Men having sex with men. Data regarding MSM are available only for 2017 and 2018.

The majority of the reported cases were males in all population groups with the exception of Roma where the two genders were almost equally affected (Table 2).

During the study period, 112 travel-related cases were reported amongst which 28 were VFRs. The majority of the travel-related cases belonged to the age group 25–44 years (56.1%) while a high percentage (46.4%) of cases among VFRs regarded children <15 years of age (Fig. 1). Overall, 22 cases (2.2%) were patients with a chronic liver disease; 18 men and four women with a median age of 37 years (3–56).

## Clusters/outbreaks 2009–2018

In the decade following the implementation of universal childhood vaccination in the country, 50 HAV clusters were recorded; 44 (with 2–12 cases, each) attributed to person-to-person transmission (24 in the general population and 20 among Roma) and six to food consumption (with 2–3 cases, each). In specific, shellfish and fish products bought from street vendors or fished from waters where fishing was forbidden were identified as the possible vehicles of the disease.

Three large HAV outbreaks were identified; in 2013 among Roma, in 2016 among refugees and in 2017 among MSM. The three outbreaks accounted for 32.3% of the total number of reported cases in the country.

In 2013, 101 HAV cases (sub-genotype IA) were reported among Roma and three distinct clusters were identified [6]. Following the 2013 outbreak, the number of recorded cases in the Roma population substantially dropped with one recorded case in the period 2016–2018.

From April to December 2016, 188 HAV cases (sub-genotype IB) with a median age of 7 years (1–29) were recorded in the childhood refugee population of the country leading to a large outbreak [9]. Cases continued to be recorded among refugees in 2017 (26 cases) and 2018 (46 cases). Reactive immunisation strategies were implemented in response and recommendations were given for the improvement of living conditions and hygiene measures inside accommodation camps as well as in reception and identification centres on the islands of the Northeastern Aegean [9].

In 2017, 294 HAV cases were reported, the highest number of cases ever recorded in the country. This increase did not regard a specific geographic region of the country. Overall, the median age of the cases was 38 years (0–70) and 240 (81.7%) of them were men. Thirty cases were migrants, 26 cases reported travel to an endemic country during the incubation period and one was Roma. Among the remaining 237 cases of the general population, 96 male cases identified themselves as MSM (21–55 years of age), 39 had unknown MSM status (contact was not possible or patients refused to answer) (21–57 years of age), 66 cases identified themselves as not being MSM (19–70 years of age) and 36 were females (17–69 years of age).

Of the 30 cases where molecular testing was applied, 23 belonged to the three co-circulating HAV strains of genotype IA of the concurrent European outbreak [4]; VRD_521_2016 (19 cases), V16-25801 (two cases) and RIVM-HAV16-090 (two cases). During the outbreak, 33 of the 96 (34.4%) male cases that identified themselves as MSM were HIV-positive. In 2018 and after the termination of the outbreak, 17 (15.5%) cases identified themselves as MSM. Management of this outbreak mainly focused on providing information to the MSM population and giving advice regarding the modes of transmission and preventive measures.

## Discussion

Detailed analysis of the available epidemiological data of symptomatic cases can be a useful tool for guiding public health action in Greece as a nationwide vaccination registry and data regarding seroprevelance in the general population and among high-risk groups of the population are not available. Published studies are dealing mostly with schoolchildren population in the country and provide some data on the vaccination coverage against HAV by age group while data on vaccination coverage over time are missing [10, 11].

Surveillance data indicated that despite the decreasing trend of HAV notification rate over the years, outbreaks continue to occur [6] providing evidence that HAV disease is a continuous public health issue in the country.

Each one of the recorded outbreaks adds evidence regarding the population groups at risk for acquiring the disease. Based on the available data for 2009–2018, two distinct epidemiological patterns were depicted; one among vulnerable populations, such as Roma and refugees with cases mainly occurring among children and one among susceptible adults.

Roma people represent the largest ethnic minority in Europe with an estimated number of 11 million dispersed throughout Europe [12]. Roma children have been, historically, the most vulnerable population group for acquiring vaccine-preventable diseases in Greece [7, 13]. Based on a vaccination coverage survey carried out at a national level in 2012–2013, coverage among Roma children aged 24–77 months was very low for established vaccines, such as DTP, IPV and MMR and even lower for vaccines introduced later in the National Childhood Immunisation Program. The vaccination coverage for the first dose of the vaccine against HAV was 22.6% (95% CI 16.4–30.2) [14]. Very low vaccination coverage of Roma children is also a consistent finding in other countries [15, 16]. Even though only one HAV case was recorded in 2016–2018 period in Greece in the Roma population, a future HAV outbreak in this population is highly likely. Following the 2013 outbreak, there has not been a systematic vaccination of Roma children against HAV, and at the same time, no data support a possible improvement of sanitation and hygiene practices inside Roma camps. Implementation of vaccination programmes specially designed for Roma populations is recommended.

Another population that was recently affected in Greece was Syrian children that entered the country in 2016. Data from other countries have shown that the childhood population of refugees from Syria is a group with low vaccination coverage, without prior infection, thus susceptible to the occurrence of HAV [17]. HAV outbreaks among Syrian refugees were also documented in other reception countries, such as Germany, Turkey and Lebanon [18–20]. Provision of proper sanitary and living conditions at accommodation camps and reception and identification centres and universal vaccination of refugee children against HAV soon after their arrival at the country should be a priority.

On the other hand, the outbreak among MSM in 2017, the first one ever documented in Greece, highlighted a different epidemiological pattern than the one described above. Even though the Greek immunisation programme includes the recommendation for vaccination of MSM, this outbreak revealed a lack of knowledge of this population regarding the transmission of the disease and the necessary preventive measures. Based on the literature, HAV outbreaks among MSM have been increasingly documented in Europe [21, 22] and the documented outbreak in 2017 in Greece was actually part of a wide European outbreak [4] where participation at national and international lesbian, gay, bisexual and transgender pride festivals was considered as a contributing factor for the occurrence and spread of the disease [23, 24]. Providing advice to this population regarding vaccination is essential. Participants at mass gathering events should be aware of sexual practices associated with HAV transmission (i.e. oral–anal, digital–anal sex) and make sure their vaccination is up-to-date.

The MSM outbreak also revealed the need for a better recording of MSM status of HAV cases in Greece and indicated that age-specific patterns of the disease have been shifted to include an increasing proportion of susceptible adults compared to previous decades, when the disease mainly concerned children. This phenomenon, also known as ‘cohort effect’, has been reported in the literature after the implementation of mass childhood vaccination and after the improvement of socio-economic conditions leading at decreased infection rates during childhood [25–27]. This ‘cohort’ effect is also depicted in our data from the increasing mean age of cases in the general population throughout the years.

Furthermore, the presented data emphasise the need for the implementation of measures for the prevention of travel-related cases. Travel is a major risk factor for HAV disease in the EU/EEA [28]. Even though the number of travel-related cases is low in Greece, pre-travel vaccination advice is probably insufficient based on the recorded data. Children and young adults that visit friends and relatives in their ancestral countries should be prioritised for vaccination, as has also been reported by other European countries [28, 29].

In addition, even though in practice people living with HIV are advised to get vaccinated against HAV, data show that vaccination in several cases is neglected. Efforts to increase vaccination coverage of this population group should be a priority since HAV disease may lead to prolonged hospitalisation and to serious complications, including fulminant hepatitis [30]. Also, HAV disease among persons living with HIV leads to prolonged viraemia and shedding, and thus to a longer infectious period [31]. Studies have shown that vaccination against HAV among people living with HIV in Greece is insufficiently low [32, 33], as also reported by other countries [34, 35].

Last but not least, the occurrence of HAV cases among people with a chronic liver disease depict that wider dissemination of clinical guidelines, and clear messaging about the safety and effectiveness of vaccination are needed. A similar need has been stated in the literature since immunisation HAV rates remain subpar in other countries too [36].

A parallel interesting finding was that the proportion of hospitalisations was unexpectedly high for such a benign disease. We can assume that there might be an overuse of hospitalisation. It is a common practice that when the cases are related to susceptible populations, such as Roma and refugees, clinical doctors choose to hospitalise the cases in order to prevent further transmission in the population. This was prominent during the 2016 outbreak that mainly involved refugee children living in hosting camps. Moreover, HAV infection is a rare disease in Greece and it is possible that clinical doctors choose to admit cases to the hospital in order to provide the appropriate follow-up. On the other hand, it is also expected that mild cases are not diagnosed or if diagnosed in the community are not always notified. In several cases when diagnosis comes without hospitalisation, clinical doctors do not report the case via the notification system. Still, evidence is not available to verify if the aforementioned hypotheses regarding the explanation of the high hospitalisation rate are correct or not and to what extent.

Overall, even though asymptomatic infections are not systematically recorded and infection rate in the country cannot be accurately estimated, epidemiological data of the symptomatic cases can offer useful insight on the evolution of the disease epidemiology in the country and can be used for the prioritisation of future public health strategies. Improving living conditions and vaccination coverage of deprived childhood population, informing young adults on the modes of transmission of the disease focusing at sexual transmission and travel and promoting vaccination of high-risk groups of the population should be the pillars of the future public health actions regarding HAV in Greece. Catch-up vaccination of young adults could be discussed in the light of available seroepidemiological data.

## Supplementary material

For supplementary material accompanying this paper visit https://doi.org/10.1017/S0950268820000382. click here to view supplementary material
