# Supplementary material for: Epidemiology of hepatitis A in Greece in the last decade: management of reported cases and outbreaks and lessons learned
Source: Epidemiol Infect. 2020 Feb 13;148:e58. doi: 10.1017/S0950268820000382 (PMC7078582; doi:10.1017/S0950268820000382)
Supplement: Supplementary file 1 [file S0950268820000382sup001.docx]

**Supplementary Table.** Number of travel related cases of hepatitis A and number of cases among travelers to country of origin (VFRs), by year in Greece, Mandatory Notification System, 2009-2018.

| **Year** | **Travel-related cases** | **Country of infection** | **VFRs** | **Country of infection** |
| --- | --- | --- | --- | --- |
| 2009 | 4 | Angola, Jordan, Kazakhstan, Russia | 4 | Lebanon, Nepal, Romania, Uzbekistan |
| 2010 | 1 | Netherlands | 1 | Bulgaria |
| 2011 | 2 | Georgia, United Kingdom | 5 | Afghanistan, Albania, Bulgaria (2), Syria |
| 2012 | 5 | Bulgaria, Germany, Madagascar, Pakistan, Uzbekistan | 3 | Afghanistan, Bulgaria, Romania |
| 2013 | 4 | Austria, Turkey (3) | 4 | Albania, Bulgaria, Netherlands, Romania |
| 2014 | 4 | Turkey (2), Seychelles, Thailand | 1 | Albania |
| 2015 | 8 | Turkey (2), Cyprus, Egypt, Romania, Spain, Sudan, unknown (1) | 3 | Albania (2), Venezuela |
| 2016 | 4 | Jordan, Ukraine, Albania, Canada | 3 | Bulgaria (3) |
| 2017 | 39* | Italy (6), Germany (3), United Kingdom (3), France (2), Netherlands (2), USA (2), Egypt (2), India (2), Bulgaria (2), Belgium, Bosnia, Brazil, Cyprus, Czech Republic, Germany, Iran, S. Africa, Spain, Thailand, Turkey, Vietnam, Former Yugoslav Republic of Macedonia, Serbia, Turkey | 2 | Albania, Bulgaria |
| 2018 | 13 | Turkey (3), Italy (2), Austria, Australia, Belgium, Morocco, Serbia, Thailand, unknown (2) | 2 | Bulgaria (2) |

* 15 of the travelers identified themselves as MSM, 12 as non-MSM, for 7 male cases MSM history was unknown and 5 cases were females.
